# A fuzzy Bayesian approach to flood frequency estimation with imprecise historical information

**DOI:** 10.1002/2016WR019177

**Published:** 2016-09-09

**Authors:** José Luis Salinas, Andrea Kiss, Alberto Viglione, Reinhard Viertl, Günter Blöschl

**Affiliations:** ^1^ Institute of Hydraulic Engineering and Water Resources ManagementVienna University of TechnologyViennaAustria; ^2^ Centre for Water Resource Systems, Vienna University of TechnologyViennaAustria; ^3^ Institute of Statistics and Mathematical Methods in Economics, Vienna University of TechnologyViennaAustria

**Keywords:** fuzzy, Bayesian, historical, flood frequency, estimation, floods

## Abstract

This paper presents a novel framework that links imprecision (through a fuzzy approach) and stochastic uncertainty (through a Bayesian approach) in estimating flood probabilities from historical flood information and systematic flood discharge data. The method exploits the linguistic characteristics of historical source material to construct membership functions, which may be wider or narrower, depending on the vagueness of the statements. The membership functions are either included in the prior distribution or the likelihood function to obtain a fuzzy version of the flood frequency curve. The viability of the approach is demonstrated by three case studies that differ in terms of their hydromorphological conditions (from an Alpine river with bedrock profile to a flat lowland river with extensive flood plains) and historical source material (including narratives, town and county meeting protocols, flood marks and damage accounts). The case studies are presented in order of increasing fuzziness (the Rhine at Basel, Switzerland; the Werra at Meiningen, Germany; and the Tisza at Szeged, Hungary). Incorporating imprecise historical information is found to reduce the range between the 5% and 95% Bayesian credibility bounds of the 100 year floods by 45% and 61% for the Rhine and Werra case studies, respectively. The strengths and limitations of the framework are discussed relative to alternative (non‐fuzzy) methods. The fuzzy Bayesian inference framework provides a flexible methodology that fits the imprecise nature of linguistic information on historical floods as available in historical written documentation.

## Key Points

- A novel framework of flood probability estimation accounting for both imprecision and stochastic uncertainty of flood information
- Imprecise (fuzzy) information on historical floods reduces the uncertainty of 100 year flood estimates by 45–61% in the case studies presented
- The fuzzy Bayesian inference framework perfectly fits the nonprecise nature of linguistic information on historical floods from archives

## 1 Introduction

Numerous extreme floods around the world in the last decades [*Hall et al*., 2014] have resulted in a renewed interest in historical floods. Due to the rich historical material, Europe lends itself particularly well to studying historical floods [see e.g., *Brázdil et al*., 2006, 2012; *Glaser et al*., 2010; *Hall et al*., 2014].

Historical floods are not only interesting from a process perspective, they can also be very useful for flood risk assessment since their record lengths are often much longer than those of the systematic data of the instrumental period [*Kjeldsen et al*., 2014]. Numerous formal statistical methods have been proposed that combine information on historical floods with systematic flood discharge measurements [e.g., *Leese*, 1973; *Cohn et al*., 1997; *O'Connell et al*., 2002; *England et al*., 2003; *Benito and Thorndycraft*, 2005] and with regional and process information [see e.g., *Merz and Blöschl*, 2008a, 2008b; *Viglione et al*., 2013]. The combination is often framed in Bayesian terms in order to estimate flood discharge probabilities and their uncertainties [e.g., *Stedinger and Cohn*, 1986; *O'Connell et al*., 2002; *Parent and Bernier*, 2003; *Reis and Stedinger*, 2005; *Neppel et al*., 2010; *Payrastre et al*., 2011].

Information on historical floods, usually, is not only stochastically uncertain but also vague or imprecise. There is an important distinction between stochastic uncertainty and imprecision. Stochastic uncertainty relates to a lack of information about the world and is usually represented by random variables. In the case of floods, the randomness represents the lack of knowledge of the timing future floods of a given discharge will occur (as represented by the flood frequency distribution), and the parameters of that distribution can be uncertain too (due to limited flood record lengths and measurement errors). Imprecision, in contrast, relates to the content of the statement on floods. For example, if historical records described a flood as a “large flood”, there is nothing uncertain about this statement. Rather it is a vague or imprecise statement, as “large” can imply a wide range of water levels. Nevertheless, this kind of information can be useful.

Fuzzy set theory [*Zadeh*, 1965] has been developed in order to represent such vagueness in a quantitative way. At the core of the theory is the membership function that defines the degree of an element's membership in a fuzzy set. A membership function of flood discharges associated with the term “large flood”, for example, represents the degree to which one thinks a particular flood discharge would be considered a “large flood.” Since the association of a given flood discharge with the term “large flood” can be partly true and partly false at the same time, the discharges are represented as fuzzy or imprecise numbers.

Fuzzy sets have been extensively used in hydrology and water resources planning in such diverse areas as geostatistics [*Bárdossy et al*., 1990], modeling water flow in the unsaturated zone [*Schulz and Huwe*, 1997], catchment water balance modeling [*Nasseri et al*., 2014], flood forecasting [*Bárdossy*, 2008], and flood polder planning [*Schumann and Nijssen*, 2011]. Surprisingly, fuzzy sets have hardly been used for quantifying historical floods even though much of the historical information is linguistic and the original development of fuzzy set theory was largely motivated by quantifying the vagueness of the human language such as the “the class of tall men” [*Zadeh*, 1965, p. 1]. We believe that the descriptions of historical floods as available in archives and other historical sources perfectly fit the nature of fuzzy modeling due to its ability to process quantitatively imprecise linguistic linguistic expressions and transform them into fuzzy numbers.

The aim of this paper is to propose a new framework that exploits the benefits of fuzzy numbers in quantifying imprecise, linguistic information on historical floods and combines them with systematic flood discharge data by a Bayesian approach. We thus link imprecision (through a fuzzy approach) with stochastic uncertainty (through a Bayesian approach) in estimating flood probabilities.

Section 2 of this paper describes typical source material of historical floods, highlighting its imprecise nature. Section 3 proposes the framework for linking specific information about historical floods with their fuzzy model, building on the fuzzy Bayesian inference theory of *Viertl* [2008a, 2008b]. Section 4 presents three case studies to illustrate the feasibility of the framework for different characteristics of the historical source material. Section 5 discusses the main findings and section 6 presents the conclusions.

## 2 Historical Data are Fuzzy

In this paper, we refer to data from the preinstrumental period as the historical time series, as opposed to data from the systematically measured period that are referred to as the systematic or instrumental time series. Historical time series may be developed from various types of written documentary evidence such as narratives (e.g., chronicles, annals, diaries), institutional sources which can be economic‐administrative (accounts) and legal‐administrative (e.g., charters, official/administrative letters, notes, reports), epigraphic evidence (e.g., flood marks, paintings/drawings), and media information (e.g., newspapers, pamphlets) etc. Depending on source availability, the quality and the regularity of observations and the recording practices, flood series may be built from one source type (e.g., accounts, flood marks, charters/letters etc.) or, more often, from a combination of various source types (e.g., narratives, epigraphic and institutional sources).

A representative example of the fuzzy character of historical documentary evidence is the general terminology applied. In medieval and early modern times in Europe, texts are often written in Latin where the overall magnitude of a flood is represented by specific terms. A clear term for an extreme flood of exceptional magnitude is deluge (“diluvium”). This term is rarely used before the beginning of the 14^th^ century, and typically used for the most extreme cases such as the floods that occurred in 1315, 1342, 1343, 1374 or 1501 in central Europe. For the Thüringen rivers in Germany, “Diluvium Thuringiacum” in Latin or “Thüringishe Sintflut” in German were used [see *Deutsch and Pörtge*, 2003]. A very large flood is sometimes referred to as “inundatio maxima” (enormous flood), for example, the 1343 Upper‐Rhine flood (Generallandesarchiv Karlsruhe Urk. 1345, Sept. 30, GLA. 16/97, Konv. 22), or “maximae aquarum inundationes” (enormous floods of water) (e.g., February 1342, by Franciscus Pragensis: *Loserth* [1875]). A large flood that is, however, not extraordinary in magnitude is usually referred to as “Nimia/magna/ingens inundatio aquarum” in Latin or “gross güß” in German, a terminology widely used in all document types (e.g., annals, chronicles, accounts, charters, diaries etc.–see e.g., *Rohr* [2004]; *Kiss* [2007, 2009]; *Kiss and Laszlovszky* [2013]; *Wetter et al*. [2011]). Some chronicles, such as the Petrak‐chronicle written in Szentes (45 km north of Szeged, Hungary), furnish a direct comparative terminology for all flood classes: “igen nagy, pusztito arviz volt” (there was a very great/destructive flood), “nagy arviz volt majusban” (there was a great flood in May) and “Ebben az evben is volt arviz” (there was a flood also in this year). *Rohr* [2004] provides a more detailed terminological discussion and other examples.

An alternative way of flood characterization are basic damage measures (e.g., casualties, buildings and infrastructure destroyed etc.), such as reported for the exceptional event in summer 1275: “Festo Petri et Pauli Rhenus pontem Basiliensem destruxit, submersis plus minus 100 hominibus.” (On the day of Peter and Paul (29 June in Julian Cal.) the bridge of Basel was destroyed, and around 100 people submerged) (source published in Latin: *Pertz* [1861]). There is thus no precise information on the maximum water levels.

Floods are also sometimes assessed with reference to human memory. Examples for unprecedented floods are “de a memoria hominum, non visa non audita” (not seen or heard about in human memory (i.e., in human lifetime)) for the extreme ice jam flood of February 1775 in Budapest, as referred to in the town protocols of BudaPest (BFL Pest. IV. 1202a. 355a. 22 Feb, 1775 [see e.g., *Kiss*, 2007]). Almost the same expression was used for the catastrophic 1343 summer flood of Lake Constance (Austrian‐German‐Swiss border): “quod antea non est visum, ut antiquiores tunc temporis referebant” (reported by J. von Winterthur: *Baethgen* [1924]).

**Figure 1:**
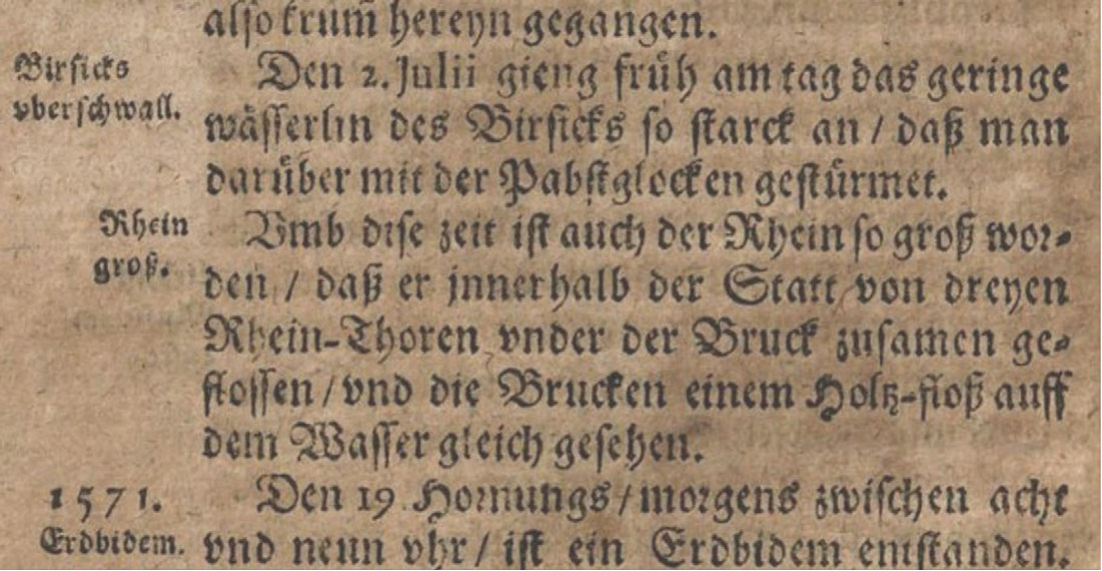
Original archival record from the “Kurtze Bassler Chronik” [*Groß*, 1624] containing the description of the 1570 Rhine flood in Basel.

Information on the extent and level of the flood waters may also appear in the source material. Examples are “die Brücken einem Holz‐floß auff dem Wasser gleich gesehen” (the bridge looked like a wooden raft on the water), referring to the 1570 Rhine flood (see Figure 1) (referenced in *Wetter et al*. [2011], taken from the Kurtze Bassler Chronik: *Groß* [1624]) and “Ganz Pest gleicht einer Insel” (the entire city looked like an island in the water), referring to the 1784 Danube flood in Budapest (*Kiss* [2007]: Pressburger Zeitung I March, 1784).

If quantitative information on water levels is available, as well as information or assumptions about the cross section, discharge can be reconstructed by hydraulic modeling [*Benito et al*., 2003; *Brázdil et al*., 2005, 2006; *Herget and Meurs*, 2010; *Wetter et al*., 2011; *Elleder et al*., 2013; *Herget et al*., 2014]. If the information is less specific regarding water tables, historical flood series are often classified into magnitude categories, and each magnitude class receives a numerical value or index. Table 1 provides the most widespread method of classifying the general information derived from the flood descriptions available in the various source types listed above. The primary indicators relate to the information that is most likely available in historical sources which is information on damage and causalities. The secondary indicators relate to potentially available hydrological information such as water tables, hydromorphological changes and the flood duration. On the basis of both indicators, floods are classified into three index classes.

**Table 1:** Typical Criteria Used in the Literature for Classifying Documentary Evidence on Historical Floods into Indices^a^

Level	Classification	Primary Indicators	Secondary Indicators
1	Smaller, regional flood	Little damage, e.g., fields and gardens close to the river, wood supplies that were stored close to the river are moved to another place	Short flooding
2	Above average, or supra‐regional flood	Damage to buildings and constructions related to the water, like dams, weirs, footbridges, bridges, and buildings close to the river, like mills, etc.; water in buildings	Flood of average duration; severe damage to fields and gardens close to the river, loss of animals and sometimes people
3	Above average, or supra‐regional flood on a disastrous scale	Severe damage to buildings and constructions related to the water, i.e., dams, weirs, footbridges, bridges, and buildings close to the river, like mills, etc.; water in buildings. In part, buildings are completely destroyed or torn away by the flood	Duration of the flood: several days or weeks; severe damage to fields and gardens close to the river, extensive loss of animals and people; morpho‐dynamic processes like sand sedimentation cause lasting damages and change the surface structure

a Taken from *Sturm et al*. [2001].

Often it is useful to prepare more specific criteria that are tailored to the characteristics of a particular case study. Table 2 shows such a tailored table for the Tisza at Szeged, Hungary. The primary indicators are more strongly based on agricultural damage, travel obstruction and flood protection issues than those in the general table. The secondary indicators use flood extent and duration instead of overbank flow because of the small elevation differences of the floodplain. For both criteria there is a clear break in 1879 because of the complete destruction of the town during the 1879 flood, followed by a fundamental change in the flood protection system of the entire river. Tables 1 and 2 summarize a set of rules that intend to minimize the effect of perception changes throughout the historical period on the classification of the magnitude of historical flood events.

**Table 2:** Criteria for Classifying the Flood Magnitudes for the River Tisza at Szeged^a^

Level	Classification	Primary Indicators	Secondary Indicators
1	Flood only slightly exceeding the limit of a usual flood event	Before 1879: Inundation area is filled up by water around the town; floodplain pastures and some cultivated fields of the town are flooded and damaged. The town is not flooded. After 1879: water more significantly exceeds the quay (low lying road along the shoreline)	Short or longer flooding; water partly exceeds the inundation area of normal flood events, but not significantly
2	Great flood, extraordinary flood	Before 1879: Cultivated lands heavily damaged. Travel is significantly obstructed. Part of the town is affected by the flood: damage to buildings/houses in some parts of the town. In the period with advanced flood protection, high alert of flood protection applied. May be combined with significant dyke breaches (but not complete dyke destruction). Neighboring settlements flooded. After 1879: water reaches significant depths over the quay, exceeds the upper section of the dykes. Buildings in the inundation area heavily flooded.	Flood of average or long duration, even months; severe damage to pasture and cultivated lands, combined with loss of animals and maybe people
3	Flood on a disastrous or catastrophic scale	Before 1879: Large part of the town severely damaged, many streets or even entire districts completely demolished, dykes severely damaged. The town has to be partly or entirely evacuated, and rebuilt after flood. Significant upgrade of flood protection system, increase of ground levels. After 1879: water is close to the crest of the dykes for several days (or even flows over them), may be combined with dyke breaches. Flood may not significantly affect the town, but ground water inundates cellars. Great damages/destruction in buildings in the inundation area. The state and army significantly intervene in flood defense.	Before 1879: Duration of flood: several weeks or longer; severe damage to the town, loss of animals and people. After 1879: flood protection on full capacity, no great damages in town.

a This is an example for a tailor‐made table of criteria for a specific case study [taken from *Kiss et al*., in prep.]. Note that the criteria change before and after 1879, as significant flood protection measures were introduced (new levee system built, and raising of the ground level close to the river).

The criteria in Tables 1 and 2 can be linked to imprecise or fuzzy numbers as will be illustrated by three case studies later in this paper.

## 3 Linking Historical Records With Fuzzy Discharges

### 3.1 Fuzzy Bayesian Inference

In this paper we propose a method that transforms the descriptions found in historical records into fuzzy peak discharges, and combines them with systematic discharge measurements by Bayesian flood frequency analysis.

Bayesian inference uses Bayes' theorem to combine prior information with observed data, in order to obtain updated information on the distribution of the parameters of a given model. For a flood frequency distribution with parameters θ, Bayes' theorem states that

(1) $$p\left(\theta |D\right)=\frac{l\left(D|\theta \right)\cdot \pi \left(\theta \right)}{\int_{\Theta }l\left(D|\theta \right)\cdot \pi \left(\theta \right)d\theta }$$

where p(θ|D) is the posterior distribution of the parameters θ, after having observed the data **D**; l(D|θ) is the likelihood function and π(θ) is the prior distribution of the parameters.

**Figure 2:**
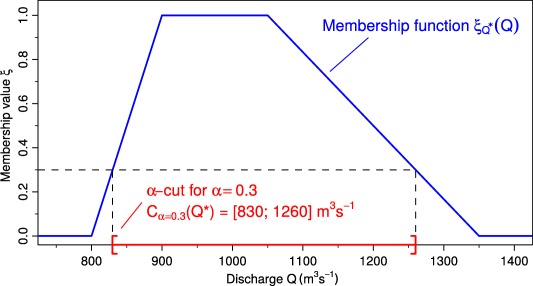
Hypothetical example of a trapezoidal membership function $\xi_{Q^{*}}\left(\cdot \right)$ associated with the fuzzy discharge $Q^{*}$. For illustration, the *α*‐cut for α=0.3 is shown as the red closed interval.

Fuzzy Bayesian Inference [*Viertl*, 2008a, 2008b] is a generalized framework for Bayesian inference when fuzzy samples $D^{*}$ and/or fuzzy prior probability distributions $\pi^{*}\left(\theta \right)$ are present, which is the case for nonprecise historical floods. Here symbols with asterisks indicate fuzzy valued functions or numbers. Fuzzy flood discharges are represented by their membership functions which give the degree of association *ξ* between discharges and a linguistic term such as “large flood” as illustrated in Figure 2. The discharge interval associated with a given membership value *ξ* is termed the *α*‐cut and the generalization to fuzzy‐valued functions is termed *α*‐levels. In this hypothetical example, a “large flood” would surely include discharges between 900 and 1050 $m^{3}s^{-1}$, corresponding to the *α*‐cut for *α* = 1, but smaller and larger discharges are more vaguely associated with the term “large flood.”

Fuzzy probability distributions are fuzzy‐valued functions with some normalizing properties, analogous to the unit integral of traditional probability distributions. Denoting the fuzzy posterior probability distribution as $p^{*}\left(\theta |D^{*}\right)$, the fuzzy‐valued likelihood of the fuzzy sample $D^{*}$ as $l^{*}\left(D^{*}|\theta \right)$, and the fuzzy prior probability distribution as $\pi^{*}\left(\theta \right)$, the generalized Bayes' theorem for their respective lower *α*‐level curves reads

(2) $${\underline{p}}_{\alpha }\left(\theta |D^{*}\right)=\frac{{\underline{l}}_{\alpha }\left(D^{*}|\theta \right)\cdot {\underline{\pi }}_{\alpha }\left(\theta \right)}{\int_{\Theta }\frac{1}{2}\left[{\underline{l}}_{\alpha }\left(D^{*}|\theta \right)\cdot {\underline{\pi }}_{\alpha }\left(\theta \right)+{\bar{l}}_{\alpha }\left(D^{*}|\theta \right)\cdot {\bar{\pi }}_{\alpha }\left(\theta \right)\right]d\theta }$$

where ${\underline{p}}_{\alpha }\left(\theta |D^{*}\right)$ is the lower *α*‐level curve for the fuzzy‐valued posterior probability $p^{*}\left(\theta |D\right),\;{\underline{l}}_{\alpha }\left(D^{*}|\theta \right)$ is the lower *α*‐level curve for the fuzzy‐valued likelihood function $l^{*}\left(D^{*}|\theta \right)$ defined in equation (3), and ${\underline{\pi }}_{\alpha }\left(\theta \right)$ is the lower *α*‐level curve for the fuzzy‐valued prior probability density function $\pi^{*}\left(\theta \right)$. The expression for the upper *α*‐level curve ${\bar{p}}_{\alpha }\left(\theta |D^{*}\right)$ is analogous, i.e., taking the upper *α*‐level curves in the numerator and keeping the same denominator. The normalizing constant in the denominator must be equal for ${\bar{p}}_{\alpha }\left(\theta |D^{*}\right)$ and ${\underline{p}}_{\alpha }\left(\theta |D^{*}\right)$, in order to keep the sequential nature of the updating procedure in Bayes' theorem [see e.g., *Viertl*, 2011a].

Since the integral in the denominator cannot be generally expressed in closed form, application of the theorem requires simulation‐based Monte Carlo sampling techniques such as the Markov chain Monte Carlo (MCMC) approach. In this paper, we use a particular variant of the MCMC method, the delayed rejection and adaptive Metropolis algorithm [*Haario et al*., 2006; *Soetaert and Petzoldt*, 2010] to obtain a given *α*‐level curve of the fuzzy posterior probability density function $p^{*}\left(\theta |D^{*}\right)$. In this paper, for clarity, the MCMC algorithm is used to estimate only the *α*‐level curves of the fuzzy posterior distribution corresponding to *α* = 0, α=0.33, α=0.66, and *α* = 1. Once the sample space of the parameters is obtained by the MCMC algorithm, we use it as an input to the inverse cumulative distribution function of peak discharges to map the uncertainty in the parameters into the credible bounds of the flood quantiles.

The fuzzy likelihood function is expressed as

(3) $$l^{*}\left(D^{*}|\theta \right)=l_{H}^{*}\left(D_{hist}^{*}|\theta \right)\cdot l_{S}\left(D_{syst}|\theta \right)$$

where $D_{hist}{}^{*}$ is the fuzzy sample containing the nonprecise historical discharges, and $D_{syst}$ is the systematic series, i.e., the discharges directly measured during the instrumental period. Note that the function $l_{H}^{*}\left(\cdot \right)$ is fuzzy‐valued, while $l_{S}\left(\cdot \right)$ is not, as the fuzziness of the systematic discharges are considered negligible as compared to the historical period.

Conceptualizing single historical events as fuzzy numbers representing the fuzzy peak discharges and merging them into a fuzzy sample, the fuzzy likelihood function for the historical discharges is simply a generalization of the non‐fuzzy case as used in the literature [see e.g., *Stedinger and Cohn*, 1986; *Neppel et al*., 2010; *Viglione et al*., 2013], which is evaluated as

(4) $$l_{H}^{*}\left(D_{hist}^{*}|\theta \right)=\left(\begin{array}{c} h\\ k \end{array}\right)F_{Q}{\left(Q_{0}|\theta \right)}^{\left(h-k\right)}\prod_{j=1}^{k}f_{Q}^{*}\left(Q_{j}^{*}|\theta \right)$$

where *h* stands for the number of years of the historical period considered, *k* is the fuzzy sample size (i.e., number of historical discharges), $F_{Q}\left(\cdot \right)$ is the cumulative distribution function of the peak discharges, *Q*_0_ is the flood perception threshold, and $f_{Q}^{*}\left(\cdot \right)$ is the probability density function for the peak discharges, taken as a fuzzy function, as it is evaluated for each historical fuzzy peak discharge $Q_{j}^{*}$. The flood perception threshold *Q*_0_, as introduced by *Francés et al*. [1994], defines the censoring discharge above which all historical flood peaks are assumed to have been recorded, while smaller floods have not. Perception thresholds are often used in flood frequency studies dealing with historical or paleoflood information, as the peak discharge time series during the historical period is rarely complete, and so is considered to be censored by an exceedance threshold (for more detail, see *Botero and Francés* [2010]).

In this paper, the Generalized Extreme Value (GEV) distribution is used as the statistical model for the annual peak discharges. Its cumulative distribution function is

(5) $$F_{Q}\left(q|\theta \right)=\text{exp}\left[-{\left(1-\theta_{3}\cdot \frac{q-\theta_{1}}{\theta_{2}}\right)}^{1/\theta_{3}}\right]$$

if $\theta_{3}\neq 0$, while the distribution converges to the Gumbel model for $\theta_{3}\to 0$. The GEV model has been chosen, as it has recently been reported to better represent the average flood regime of European rivers than alternative distributions [see e.g., *Salinas et al*., 2014a, 2014b], but any other extreme value distribution could be equally well used here. The only requirement in the model choice, related to the derivation of the informative prior distribution described in section 3.3.2, is that the flood frequency distribution should be a three‐parameter distribution.

All data (historical and systematic) are used here as annual maxima series (one value per year). In case the flood perception threshold varies during the historical period, we divide the historical period into *q* subperiods with different perception thresholds, such that $h=h_{1}+h_{2}+\ldots +h_{q}$, apply the expression for the likelihood function to each subperiod with its own perception threshold, and multiply the values of all subperiods to obtain the expression for the fuzzy likelihood function for the entire period.

The expression of $l_{S}\left(D_{syst}|\theta \right)$ does not require any special treatment, as the systematic discharges are considered as precise (i.e., non‐fuzzy), and corresponds to the product of the probability density function $f_{Q}\left(\cdot \right)$ evaluated at all measured peak discharges.

### 3.2 Design of Case Studies

To illustrate the feasibility of the approach for real world situations we present three case studies in this paper. The case studies were selected to cover a variety of hydromorphological conditions and historical source material types, and consequently different degrees of fuzziness.

The first case study is the Rhine at Basel (Switzerland), an Alpine river with a bedrock profile at Basel. This facilitates discharge reconstructions from water levels. These discharges are used along with historical narratives and systematic discharge observations. We use a noninformative prior and combine all the information in the likelihood function.

The second case study is the Werra at Meiningen (Germany), a midland river with a less well defined profile. Therefore, magnitude indices of historical floods are used instead of discharge reconstructions. The indices are incorporated in an informative prior. The likelihood function combines narratives of selected flood events through their membership functions, as well as systematic discharge observations.

The third case study is the Tisza at Szeged (Hungary), a flat lowland river with extensive flood plains. There are fewer permanent landmarks throughout the entire historical period which, together with the flat topography, contributes to a larger vagueness of the historical floods than in the other case studies. The presence of an extensive floodplain with small elevation differences around Szeged makes discharge reconstruction difficult. Therefore, no discharge observations are used here. Instead, the magnitude indices of historical floods are incorporated into the likelihood function through their membership functions in an analogous way as the discharges in the Rhine case, and a noninformative prior is used.

The type of information included in the prior distribution and the likelihood function for the three case studies is summarized in Table 3.

**Table 3:** Design of Case Studies in This Paper, Classified According to the Choice of the Likelihood Function and the Prior Distribution

Case Study	Prior Distribution	Likelihood Function
Rhine at Basel	• Noninformative, non‐fuzzy prior	• Membership functions for discharges based on historical discharge reconstruction and narratives (fuzzy) • Systematic discharge observations (non‐fuzzy)
Werra at Meiningen	• Informative fuzzy prior based on historical flood magnitude indices and discharge threshold between magnitude classes	• Membership functions for discharges based on historical narratives for selected events (fuzzy) • Systematic discharge observations (non‐fuzzy)
Tisza at Szeged	• Noninformative, non‐fuzzy prior	• Membership functions for magnitude index based on narratives (fuzzy)

### 3.3 Fuzzy Prior Distributions

#### 3.3.1 Noninformative Prior Distributions–Rhine and Tisza

For the Rhine and Tisza case studies, there is no need to specify an informative prior since both historical and systematic data can be straightforwardly included in the likelihood function. Therefore we assumed a noninformative flat prior, i.e., π(θ)∝1 for all values of θ. If additional, independent information is available, it can be easily used in an informative prior. Note that π(θ) is non‐fuzzy (precise).

#### 3.3.2 Informative Prior Distributions–Werra

For the Werra case study, we use a fuzzy valued informative prior related to the threshold discharges between the flood magnitude indices (0 to 1, 1 to 2, 2 to 3). The prior distribution is fuzzy valued because we define the censoring discharges between magnitude classes as imprecise (fuzzy) numbers. It is constructed in the following way.

**Figure 3:**
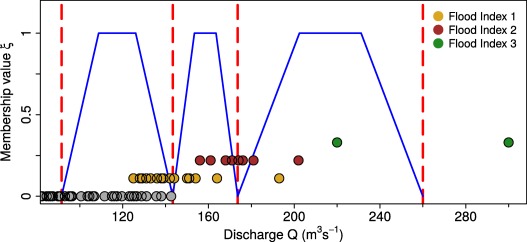
Trapezoidal membership functions (blue lines) for the three fuzzy discharge thresholds between flood indices. Data correspond to the river Werra case study. Grey, dark yellow, dark red, and green circles represent years with index values 0, 1, 2, and 3, respectively. Red dashed vertical lines represent the average discharges for each flood index.

For a stationary stochastic process, consisting of a sequence of independent and identically distributed random variables (in our case, the flood peak time series, modeled as a sequence of independent GEV realizations), the mean inter‐arrival time of events larger than a given threshold has an Inverse‐Gamma sampling distribution [*Kottegoda and Rosso*, 1997]. The estimated sample means of the inter‐arrival times between events inside each index therefore define three Inverse‐Gamma sampling distributions. Using these as marginals and a Gaussian copula for the correlation structure, we construct the trivariate distribution of the inter‐arrival times. The correlation parameter of the copula is estimated by simulations based on the systematic sample, i.e., the measured discharges during the instrumental period. This trivariate distribution can be interpreted as a trivariate sampling distribution for the three return periods associated with the three threshold (censor) discharges. The trivariate distribution of the inter‐arrival times is then transformed into a trivariate fuzzy valued distribution for the parameters of the parent GEV distribution (i.e., the prior distribution $\pi^{*}\left(\theta \right)$ as in equation (2)) based on the Jacobian of the transformation from return periods $T=\left(T_{1},T_{2},T_{3}\right)$ to parameters $\theta =\left(\theta_{1},\theta_{2},\theta_{3}\right)$, which is a function of the fuzzy valued threshold discharges. These discharges are estimated here by a simple method. Taking the annual peak discharges from the overlapping period between available flood indices and measured flows, we assign to each discharge value an index of 0, 1, 2 and 3 (an index of 0 represents years that have not been classified as 1, 2, or 3) and estimate the discharge averages for each index. These index averages are assumed to correspond to membership values of 0 of the fuzzy thresholds, and the membership function is assumed to be trapezoidal. For example, the fuzzy threshold between indices 1 and 2, as seen in Figure 3, is a trapezoidal fuzzy number whose *α*‐cut for *α* = 0 ranges from 141 $m^{3}s^{-1}$ (average discharge for flood events classified as index 1, dark yellow points in Figure 3) to 170 $m^{3}s^{-1}$ (average discharge for flood events classified as index 2, dark red points in Figure 3). The *α*‐cut for *α* = 1 is then computed as one third (lower bound) and two thirds (upper bound) of the range defined by the *α*‐cut for *α* = 0.

### 3.4 Fuzzy Likelihood Functions–Parameterization of Membership Functions

For all case studies, membership functions associated with the historical event discharges $Q_{j}^{*}$ (indices for the Tisza) were constructed from the historical descriptions. These were used in the likelihood functions. The two main characteristics of the membership functions are their location and their width. The location represents the magnitude of the floods, and the width represents the vagueness or imprecision.

#### 3.4.1 Magnitudes, i.e., Midpoints of Membership Functions

Table 4 contains, for each case study, text examples for the three magnitude categories of Tables 1 and 2. For the Rhine at Basel and the Werra at Meiningen they reflect the kind of information *Wetter et al*. [2011] and *Deutsch and Pörtge* [2003] used to assign water levels and indices, respectively. For the Tisza at Szeged, they reflect the information used in this paper to assign indices.

**Table 4:** Representative Examples From the Three Case Studies of Textual Descriptions of Flood Events Corresponding to Three Magnitude Levels (as in Table 1)

Magnitude	Event	Original Text	English Meaning
	Basel, Nov. 1445	“Am sonntag vor sant Catherinen tag vor sunnen uffgang was der Ryn grosz; für ein boum an ein Rinmúle und stiesz die múlin hinweg, und by Merckt gieng das schiff mit der müli under.” [*Bernoulli*, 1895, p. 386]	On Sunday before Saint Catherine's day, before sunrise the Rhine was large; it took a beam to the watermill and dragged away the mill, and the boat with the mill went down near the market.
small	Meiningen, Jan. 1820	“Nachdem (18. Jan.) mehrere Tage strenge Kälte gewesen war, trat Thauwetter ein und am 20. Jan. war in Folge desselben die Werra aus den Ufern getreten daß das Wasser die Chaussee am obern Rafen überstieg.” (Chronik)	After that for some days strong coldness occurred, and then melting, and as a consequence on 20 January the Werra left its bed and the water exceeded the promenade on the upper stage.
	Szeged, Nov. 1718	Visitatio in Győ, die 14. Novembr(is). “Ob impedimentum Navis pro transitu Tibisco, Praedicantem cum Judicae, et Juratis Pagi Gyö in eodem Comitatu (Csongrad) existentis ad Nos evocavimus,…”	As the ship was obstructed by the flood of the Tisza, (we did not go there, but) we invited to us the preacher with the judge and other leaders of the settlement/land Győ located in the same (Csongrad) County.
	Basel, Jul. 1511	“Anno dom. 1511. sabbato ante Mariae Magdalenae was der Rin zu Basel also groß, das in niemant in 31 jären großer verdacht,….” (Anzeiger)	In 1511 on Saturday before (the day of) Maria Magdalena the Rhine at Basel was so large that nobody has seen it larger for 31 years,…
medium	Meiningen, Jun. 1816	“Am 28. Jun. hörte es nicht auf, stark zu regnen; die Werra wuchs und nahm vieles Holz auf dem obern Rafen und die Hälfte des langen Stegs am mittleren Rafen hinweg. Viele Gärten um die Stadt standen im Wasser, auch Heu auf den Wiesen ward fortgeschwemmt.” (Chronik)	On 28 June it was heavily raining as was not heard before; the Werra increased and took much wood from the upper stage, and it took away half of the long jetty. Many gardens in the town were inundated, and also the hay on the meadow was taken by the flood.
	Szeged, Apr. 1784	Magyar Hirmondó, 28 April 1784, p. 269: “Szegedről eme' hónak tizen harmadikán (13 April) költ levélbenn írják: hogy a' Tisza kitsiny idő alatt nagy sebesen anynyira meg áradott: hogy a' szomszéd faluk, Tápé, és (Al)Győ egészen vízbe merültek. Az útakat is úgy el folyta a' víz, hogy egy helységből a' másikába szörnyő tsavargó kerülettel kelletik bé kotsizni. A' Váras mind azon által még nem áll vízbenn, egynehány házakat ki vévén, melylyek alább fekszenek szinte a' Tisza parton.”	Letter written on 13 of this month, from Szeged: that the Tisza within a short period of time rather suddenly increased so much that the neighbouring villages, namely Tápé and (Al)Győ have been entirely submerged in the water. Water washed away roads so much that a great detour has to be made to travel by coach from one settlement to another. Still, the town is not yet standing in water except for some houses, those lying lower, close to the bank of the Tisza.
	Basel, Jul. 1480	“Anno 1480 circa Magdalenae ingens fuit aquarum inundatio. Rhenus in tantum crevit, quod ad lateres usque inferioris muri Minoris Basileae circa litus ascendit. Etiam homines in ponte stantes facile in Reno manus lavare poterunt.” [*Bernoulli*, 1915, p. 210]	In 1480, around Magdalena('s day) there was a huge flood of waters. The Rhine rose to the sides of the lower walls of Lesser Basel, near the shore. People standing on the bridge could wash their hands in the Rhine.
large	Meiningen, Feb. 1784	“Am 27. Febr. brach das Eis, die Werra wurde sehr groß: das Marktwasser trat vom untern Thore herein, lief in der untern Marktgasse in die Häuser und stieg bis auf den Markt. Aus dem Marstall wurden die Pferde in die Stadt gebracht.” (Chronik)	On 27 Feb. the ice broke and the Werra was very large: the water entered through the lower Gate, poured into the houses of the lower Marktgasse and reached the market. The horses were brought into the city from the stables.
	Szeged, Feb. 1816	“… már a Város nagyobb része vízben vagyon, a mennyire a Conscriptio meg tétethetett, s‐ezúttal bé adatott 1495. alacsonyabb fekvésü házak öszve rogytak, a K. Éléstár‐is vízzel körül vagyon, a kocsival való járás sok utczákban lehetetlen, a víz eresztés, méretes szakadatlanul, de sikertelenül folytatódik, azon feölül hogy az házaikbul ki‐szoroultaknak így magasabb helyeken kell menedéket szerezni, még minden reménység csak a feö töltések meg tartásában vagyon,…”	… most parts of the Town are already in water, and when it was possible a Conscription was made and submitted: 1495 houses in lower‐lying areas have collapsed; the Royal Granary is surrounded by water, it is impossible to travel by coach in many streets, the drainage unsuccessfully continues, and beyond the fact that those lost their houses have to find refugee on higher terrain, all hope is in maintaining and supporting the main dykes,…

The first three descriptions in Table 4 (Basel 1445, Werra 1820, and Szeged 1718) correspond to flood events of a small magnitude. In all three cases, the flooding caused a disturbance in the settlement activities, economy, (e.g., “…it took a beam to the watermill…”), without specific mention of any considerable damage. This is a characteristic feature of historical floods of magnitude 1 (see Table 1), i.e., small. The following three events (Basel 1511, Werra 1816, and Szeged 1784) belong to the intermediate magnitude category. Larger disturbances than in the previous case are explicitly present in the descriptions (e.g., “…it took away half of the long jetty…”) and the flood extent is reported in a more precise way (e.g., “…the town is not yet standing in water except for some houses, those lying lower, close to the bank of the Tisza…”), as is common in the intermediate flood category. The last three descriptions in Table 4 (Basel 1480, Werra 1784, and Szeged 1816) portray large magnitude flood events. In all three texts there is mention of the extraordinary magnitudes of the flood events (e.g., “…ingens fuit aquarum inundatio/there was huge inundation of waters…”). These wordings are not usually found in this kind of descriptions unless the damage, extent and duration of the flood were considerably larger than those of previous floods.

**Figure 4:**
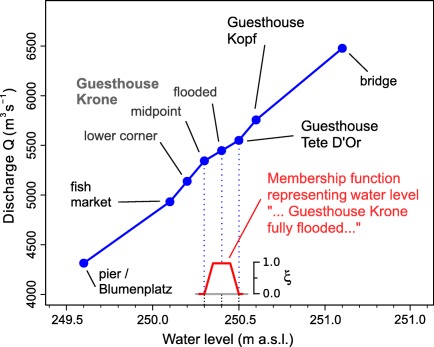
Rating curve (blue line) for the river Rhine at Basel, cross section at the Rheinbrücke (taken from *Wetter et al*. [2011]). Blue points indicate locations mentioned systematically in the documentary evidence. An example of the construction of the membership function for the fuzzy water level corresponding to the description “…Guesthouse Krone fully flooded” is depicted in red.

For the Rhine case study we used only the discharges from the reconstructions of *Wetter et al*. [2011] to determine the midpoint of the membership functions. Figure 4 illustrates their method of analyzing water levels reaching different locations in the vicinity of the river, which were systematically reported in historical documents. For example, during the 1690 flood it has reached the fish market while during the 1801 flood it has reached the Guesthouse “Tete D'Or”. Equally important is the information (or the inference) that a particular location has been reached, but the next higher location has not. For example, the 1764 event was reported to reach the lower corner of the Guesthouse “Krone”, which allowed *Wetter et al*. [2011] to infer that the building itself was not flooded, and therefore gave an indication of the maximum water level of this flood. *Wetter et al*. [2011] estimated the stage‐discharge relationship (Figure 4) by hydraulic modeling, assuming no change in the river cross section. Given that the cross section at Basel consists of bedrock this seems to be a reasonable assumption. In this paper, these discharges were assumed to correspond to the midpoints of the membership functions for the historical floods in the period 1256–1867.

For the Werra case study we used the indices presented by *Mudelsee et al*. [2006] (that are based on source material compiled by *Deutsch and Pörtge* [2003] and *Deutsch et al*. [2004]), transformed them into discharges (on the basis the measured discharges of the systematic period), and used them for the informative prior distribution. Since the cross sections are less well defined than for the Rhine, no hydraulic discharge reconstructions of historical floods were performed. In the likelihood function we only use five historical events represented by fuzzy discharges, the midpoints of which are taken as the average discharges of the index classes (red vertical lines in Figure 3).

For the Tisza case study we used directly the criteria in Table 2 to obtain the indices from source material. Due to the flat terrain of this lowland river a choice was made to perform the analysis on the basis of indices only. The integer indices were modified by a small fraction of up to 10% (e.g., from 2 to 2.1) to adapt to nuances of the narrative that provided more detail than can be captured by an integer index. These adapted flood indices were then used as the midpoints of the membership functions representing flood magnitudes.

#### 3.4.2 Vagueness, i.e., Widths of Membership Functions

The main strength of the fuzzy approach is that it is not only able to capture the magnitude of the events, but also their degree of imprecision or vagueness. Table 5 contains, for each case study, examples of textual descriptions for three degrees of imprecision. We used this kind of information to classify events of all case studies according to their level of vagueness from which we assigned the widths of the membership functions.

**Table 5:** Representative Examples From the Three Case Studies of Textual Descriptions of Flood Events Corresponding to Three Imprecision Levels

Imprecision	Event	Original Text	English Translation
	Basel, Jul. 1480	“Anno 1480 circa Magdalenae ingens fuit aquarum inundatio. Rhenus in tantum crevit, quod ad lateres usque inferioris muri Minoris Basileae circa litus ascendit. Etiam homines in ponte stantes facile in Reno manus lavare poterunt.” [*Bernoulli*, 1915, p. 210]	In 1480, around Magdalena('s day) there was a huge flood of waters. The Rhine rose to the sides of the lower walls of Lesser Basel, near the shore. People standing on the bridge could wash their hands in the Rhine.
small	Meiningen, Jan. 1820	“Nachdem (18. Jan.) mehrere Tage strenge Kälte gewesen war, trat Thauwetter ein und am 20. Jan. war in Folge desselben die Werra aus den Ufern getreten daßdas Wasser die Chaussee am obern Rafen überstieg.” (Chronik)	After that for some days strong coldness occurred, and then melting, and as a consequence on 20 January the Werra left its bed and the water exceeded the promenade on the upper stage.
	Szeged, Jul. 1816	“Alább in a Te(kinte)tes N(eme)s Tanácsnak Kegyes rendeléséhez képpest alázatossan jelentem hogy a Tisza vize, a Maros vízének szörnyü és az idein legnagyobb mostani kiöntése miáll szüntelen árad úgy hogy a múlt Julius 8ik napjátúl fogva 2 Hüvelknyire Fölmagossodott melly szerént a Tiszának az idei legnagyobb aradásátúl fogva esett apadása 20 Hüvelknyit tészen. Szeged 12ik Julii 816. Vedres István”	Letter to the Locumtenential Council: Below, following the order of the esteemed noble council, I report that the water of the Tisza, due to the horrible and in this greatest present inundation of the waters of the Maros, is constantly increasing, and so from the (past) 8 July it increased with 2 inches and thus, the total decrease of the water level of the Tisza compared to the highest levels takes 20 inches. Szeged, 12 July 1816. Istvan Vedres
	Basel, Jul. 1570	“Umb dise zeit ist auch der Rhein so großworden/daßer innerhalb der Statt von dreyen Rhein‐Thoren under der Bruck zusamen geflossen/und die Brucken einem Holz‐floßauff dem Wasser gleich gesehen.” [*Groß*, 1624, p. 210]	Around this time the Rhine was also so large that it flowed together under the bridge through the three Rhine‐gates in the downtown, and the bridge looked like a wooden raft on the river.
medium	Meiningen, Jun. 1816	“Dreimal trat die Werra aus den Ufern; der ganze Grund stand gegen 14 Tage auf dem Halme aus und die wenige Frucht, die man ärndtete und feucht einfuhr, gab schlechtes Brod.” (Chronik, p. 197)	The Werra left its banks three times; water was on the whole ground covering the stalks and the little grain people harvested, gave bad bread.
	Szeged, 1741	“… preter quod enim quod eadem exundatio agris seminaturas Prata et Pendentes in Vinejs agris fructus in Locis praeterea Marusio et Tibisco Vicinis totaliter perfuderit, et in nihilum redegerit etiam in forejs quod Miser Incola n(on) sui sustentatione conservabat inundavit et Usui humani redidit inutile” (CsCP)	…, and beyond that the same inundation completely flooded the arable lands (with crops), pastures, vineyards and orchards along the Maros and Tisza rivers, and gave nothing to people, and became useless for human use.
	Basel, 1268	“Renus crevit usque adeo, quod omnes pontes destruxit.” (Ann., p. 193)	The Rhine rose so much that it destroyed all bridges.
large	Meiningen, May 1818	“Nach anhaltenden Regenwetter trat die Werra aus den Ufern. Am 18. Mai war die Ueberschwemmung am bedeutendsten.” (Chronik, p.205)	After persistent rainy weather the Werra left its banks. The flooding was the most significant on 18 May.
	Szeged, 1731	3o. “Ioanni Hegedüs, ex quo exundatio aquarum modernam Domum residentionalem destruxisset,…” (SzCP)	The house of Janos Hegedüs has been destroyed by the recent flood,…

For the events with small imprecision (Basel 1480, Werra 1820, and Szeged 1816) there is explicit mention of the water level that was reached during the flood (e.g., “…on 20 January the Werra left its bed and the water exceeded the promenade on the upper stage…”). The water levels provided in these three descriptions are quite precise, up to the point that one could perform flood discharge reconstructions, given an elevation model at the time of the flood and some assumptions about the roughness of the terrain. The intermediate precision category (Basel 1570, Werra 1816, and Szeged 1741) also provides information about the maximum water levels, but these are given in a vaguer manner (e.g., “…the bridge looked like a wooden raft on the river…”), and there is a stronger focus on the flood extent (e.g., “…water was on the whole ground covering the stalks and the little grain…” or “…the same inundation completely flooded the arable lands […], pastures, vineyards and orchards…”). These pieces of information will result in wider membership functions. In the large imprecision examples (Basel 1268, Werra 1818, and Szeged 1731) there is no mention of water levels or the spatial extent of the flooding. There is a vague mention of large damages (e.g., “…it destroyed all bridges…” or “…the house of Janos Hegedüs has been destroyed…”).

**Figure 5:**
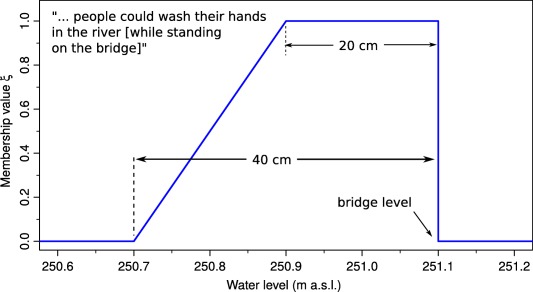
Trapezoidal membership function (blue line), representing the fuzzy water level for the flood event of 1424 in Basel, described as “…people could wash their hands in the river…” in the chronicles of Kaplan Hieronymus Brilinger [*Bernoulli*, 1915].

For the Rhine case study, there are two groups of events with different types of information. The first group consisted of floods where we derived the vagueness from the spatial extent of the locations (e.g., market square) mentioned, and Table 5 was not used. A membership value of 0 was given to the water levels of the previous and following locations to the one described as flooded as illustrated in Figure 4 where the imprecision associated with the water level of 250.4 (Guesthouse Krone fully flooded) ranges from 250.3 (Guesthouse Krone, centre of building) to 250.5m (Guesthouse Tete D'Or'). Membership values of 1 were given within half of the water level ranges between locations. The second group of events were floods where such information on the extent was not available. For these floods we directly used the linguistic information as of Table 5. For some of these floods clear information about the vagueness was available. For example, the description of the 1424 flood has “… people could wash their hands in the river” (Figure 5). On the one hand, the peak water level most likely did not exceed the level of the bridge, as this would have been reported. On the other hand, if people could wash their hands in the river while standing on the bridge, the water level could not have been lower than 20–40 cm below the level of the bridge itself. While the quote represented in Figure 5 may sound like a hyperbole, it should be noted that the historical sources used in this study were contemporary and written by local reliable authors, which suggests that the description can be taken in a literal sense. Some minor changes probably occurred over time, and these are accounted for by the membership function.

For other floods the descriptions were less clear. For example, the description of the 1570 flood has “…boats were boarded through the windows of the Guildhouse”. For such events the vagueness was grouped according to Table 5. The widths were selected as 3, 6, 9% of the midpoint (*α*‐cut of 1) and 10, 15, 20% of the midpoint (*α*‐cut of 0) (in terms of discharges), for small, medium and large imprecision, respectively. The membership function was assumed to be trapezoidal with a few exceptions (e.g., 1268) where the upper limb was assumed to consist of two linear sections reflecting detailed historical descriptions. All water levels were transformed to discharges by the same rating curve as the midpoints.

For the Werra case study, Table 5 was always used for the selected five events. The widths were selected as 3, 6, 9% of the midpoint (*α*‐cut of 1) and 10, 15, 20% of the midpoint (*α*‐cut of 0) (in terms of discharges), for small, medium and large imprecision, respectively. The membership function was assumed to be trapezoidal.

For the Tisza case study, Table 5 was used for all events. The widths were selected as 3, 6, 9% of the midpoint (*α*‐cut of 1) and 10, 15, 20% of the midpoint (*α*‐cut of 0) (in terms of indices), for small, medium and large imprecision, respectively. The membership function was assumed to be trapezoidal.

## 4 Case Studies

### 4.1 Reconstruction Based on Maximum Water Level Descriptions Since 1256 (Rhine at Basel, Switzerland)

The Rhine at Basel drains an area of about 36,000 km^2^, and elevations range from 244 to 4274 m. Floods typically occur in late spring and summer as a result of extensive rainfall of 1 or 2 days. Snowmelt may increase the antecedent soil moisture [*Scherrer et al*., 2008].

**Figure 6:**
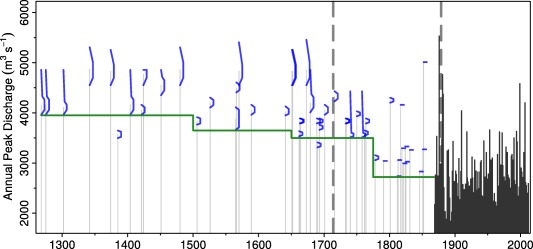
Fuzzy sample of the historical floods in the period 1256–1867 (membership functions in blue) and systematic flood peak discharges (black), Rhine at Basel. The green line indicates the perception threshold, the vertical grey lines indicate years of diversion.

The historical flood discharge series of *Wetter et al*. [2011] used here contains 36 events from the period 1256–1867, making up the fuzzy sample. Additionally, 142 measured annual peak discharges from the instrumental period (1869–2010) are used which make up the non‐fuzzy sample. As noted in section 3.4.1, the rating curve shown in Figure 4 is taken as constant during the entire historical period. This assumption is supported by the local geology of the river bed along the city of Basel, which mainly consists of bedrock [*Wetter et al*., 2011]. Two important river diversions reducing the flood magnitudes took place in 1714 and 1877. *Wetter et al*. [2011] estimated the peak discharge reductions as 630 and 900 $m^{3}s^{-1}$ for the first and second diversion, respectively. In this paper we harmonized the entire time series to present day conditions by subtracting the estimated peak discharge reductions in the corresponding periods (Figure 6). During the historical period the flood perception threshold is considered to have changed three times, given the evidence presented in *Wetter et al*. [2011] (in 1500, 1650, and 1780), and in each subperiod the perception threshold is taken as the lower bound of the smallest fuzzy discharge (5611, 5261, 4816, and 4300 $m^{3}s^{-1}$) (with two exceptions which were considered not typical).

**Figure 7:**
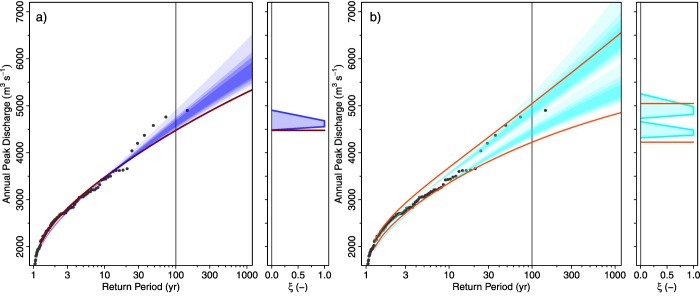
Flood frequency curves for the Rhine at Basel. Points are the observed discharges (systematic sample). Red lines are estimates from the systematic sample alone. Shaded areas are the fuzzy estimates obtained by combining the fuzzy historical sample and the systematic sample. The color intensities of the shaded areas represent the *α*‐levels of the fuzzy flood frequency curves for *α* = 0, α=0.33, α=0.66, and *α* = 1. (a) The posterior mode estimates and (b) the 5% and 95% credibility bounds.

Figure 7 shows the estimated fuzzy flood frequency curve (*α*‐level curves represented by blue transparent polygons) in panel (a) and its fuzzy 5% and 95% Bayesian credibility bounds (cyan) in panel (b). As compared to the non‐fuzzy estimates from the systematic data alone (red line), the discharges associated with large return periods are somewhat higher. The imprecise 100 year flood discharge (without stochastic uncertainty) (*α*‐cut for *α* = 0) is 4483–4905 $m^{3}s^{-1}$ while the estimate from the systematic data alone is 4480 $m^{3}s^{-1}$. The steeper tail is more consistent with the largest measured flood discharges (points in Figure 7) which is a reflection of the additional information obtained from the historical data. Based on a combination of rainfall runoff modeling and flood statistics *Scherrer et al*. [2008] suggested that a discharge of 5000 $m^{3}s^{-1}$ in Basel is associated with a return period between 100 and 300 years. This is similar to the range that is obtained in this study for the imprecision (without stochastic uncertainty) of that discharge for an *α*‐cut for *α* = 1 (120–360 years) (Figure 7a). It is interesting that the fuzziness is small at small return periods and increases substantially as the return period increases. This behavior is related to the choice of a non‐fuzzy (precise) prior distribution which, in combination with the non‐fuzzy (precise) systematic data, controls the fuzziness of the small return periods. For larger return periods, the fuzzy component of the likelihood function associated with the historical events becomes more important.

Figure 7b shows that the imprecise credibility bounds have a similar imprecision as the best estimates in Figure 7a. If one compares the midpoints of the estimated membership functions of the credibility bounds with their non‐fuzzy counterparts from the systematic data alone, there is a significant reduction in the width of the bounds resulting from the use of historical information which is not surprising given that the historical sample covers 612 years. However, this is at the cost of introducing imprecision.

### 4.2 500 Year Series Based on Mixed Source Evidence (Werra at Meiningen, Germany)

The Werra at Meiningen drains an area of about 1170 km^2^, and elevations range from 282 to 982 m. Floods may occur throughout the year but large winter floods are more frequent, often associated with rain‐on‐snow, and sometimes with ice jams.

**Figure 8:**
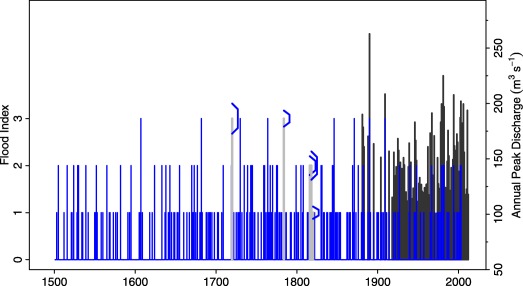
Fuzzy sample of the historical indexed floods in the period 1500–2003 (blue) and systematic flood peak discharges (black), Werra at Meiningen. Membership functions of the five selected events are shown.

The historical flood magnitude index series published by *Mudelsee et al*. [2006] used here is based on various types of local sources (e.g., chronicles, annals, diaries, newspapers, pamphlets etc.). It contains 128 indexed floods from the period 1500–2003 (Figure 8) which were used here to estimate the fuzzy prior distribution. On the basis of an assessment of the source material a judgment was made that approximately 20% of index 1 floods were not identified. Therefore, 20% of the years with index 0 (no flood observed) were randomly assigned index 1 (small flood observed). Additionally, the membership functions of five historical events (1720, 1784, 1816, 1818, and 1820) were used in the likelihood function along with (non‐fuzzy) measured annual peak discharges from the instrumental period (1918–2012). The information contained in the membership functions of the five historical events is assumed to be independent from the inter‐arrival times estimated from the 128 indexed floods.

**Figure 9:**
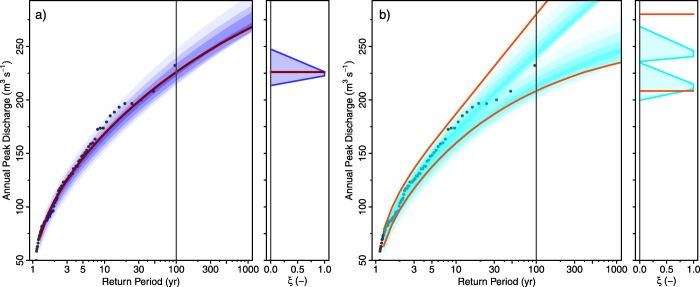
Flood frequency curves for the Werra at Meiningen. Points are the observed discharges (systematic sample). Red lines are estimates from the systematic sample alone. Shaded areas are the fuzzy estimates obtained by combining the fuzzy historical sample and the systematic sample. The color intensities of the shaded areas represent the *α*‐levels of the fuzzy flood frequency curves for *α* = 0, α=0.33, α=0.66, and *α* = 1. (a) The posterior mode estimates and (b) the 5% and 95% credibility bounds.

Figure 9 shows the results of the analysis. The fuzzy estimates (blue polygons in Figure 9a) are quite consistent with the non‐fuzzy estimates from the systematic data alone (red line). The imprecise 100 year flood discharge (without stochastic uncertainty) (*α*‐cut for *α* = 0) is 213 – 245 $m^{3}s^{-1}$ and the estimate from the systematic data alone is 227 $m^{3}s^{-1}$. It is interesting that the imprecision grows less with the return period than for the Rhine case study. This is because a fuzzy prior distribution is assumed here which propagates to the fuzziness of all return periods to a similar extent. The membership functions of the estimates are almost triangular which is a reflection of the shape of the membership functions of the threshold discharges. As the discharges are derived from the thresholds *between* the indices, the range of the *α*‐cut for *α* = 1 becomes narrow.

Figure 9b shows that the imprecise credibility bounds are slightly overlapping but the *α*‐cuts for *α* = 1 are quite distinct. If one compares the midpoints of the estimated membership functions of the credibility bounds with their non‐fuzzy counterparts from the systematic data alone, there is a clear reduction in the width of the bounds resulting from the use of historical information.

### 4.3 Combination of Flood Indices and Contemporary Institutional Source Evidence (Tisza at Szeged, Hungary)

The Tisza at Szeged drains an area of about 157,200 km^2^, and elevations range from 79 to 2500 m (median of 220 m). Floods tend to occur from spring to summer, and some floods may linger around in Szeged for months because of the extremely flat terrain. The flat terrain also contributes to the greater vagueness of the historical information than in the other case studies. Compilation of the historical flood index series of Szeged started from documentary evidence collected from the local historical literature which was then double checked against original sources (e.g., administrative, private correspondence and newspapers). Additionally, continuous information was collected from the systematic protocols of the Szeged town council, and those of the county meetings [*HNA‐CsML*, 1849], and/or in general town and county documentation such as tax release records, and sometimes also in the form of separate archival fonds on floods (e.g., 1816 or the 1879 floods: *HNA‐CsML* [1819, 1879]). From 1833 daily water level measurements and descriptions of dike breaches were used to check the index classification. To complement this information, some narratives from the Szeged region were used.

**Figure 10:**
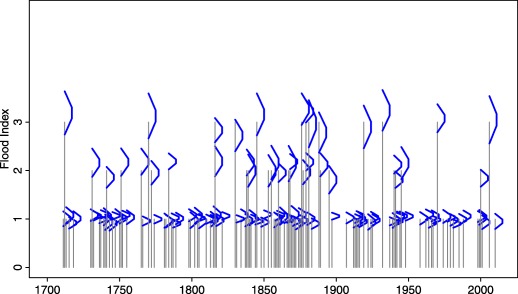
Fuzzy sample of the historical indexed floods in the period 1700–2005 (blue), Tisza at Szeged.

The index classification is based on Table 2. Indices were preferred for the series over explicit water levels due to river training measures, morphological changes and the unavailability of water levels before 1833. Although presumably all extreme and catastrophic flood events were documented, the detection of index 1 flood events is probably not systematic until the early 19^th^ century. Similar to the Werra case study, a judgment was made that approximately 20% of index 1 floods were not identified and 20% of the years with index 0 where randomly assigned index 1. The length of the historical flood series is limited by the beginning of the 18^th^ century due to the wars associated with the end of the Ottoman occupation, so the record is from 1700 to 2005 (Figure 10).

**Figure 11:**
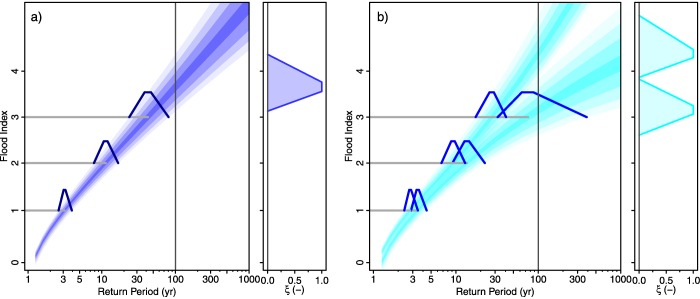
Magnitude frequency curves for the Tisza at Szeged. The color intensities of the shaded areas represent the *α*‐levels of the fuzzy flood frequency curves for *α* = 0, α=0.33, α=0.66, and *α* = 1. (a) The fuzzy posterior mode estimates and (b) the fuzzy 5% and 95% credibility bounds. Membership functions of the plotting positions associated with the indices are shown.

Figure 11 shows the results of the analysis in terms of imprecise magnitude frequency curves. The imprecise 100 year flood index (without stochastic uncertainty) (*α*‐cut for *α* = 0) is 3.15 ‐ 4.32. While the indices of Table 2 have only been specified for a range from 1 to 3, larger events are possible. This is because Table 2 relates to the sample while the estimates in Figure 11 relate to possible future floods that may be bigger than any flood that has been observed in the past. The vagueness (or imprecision) of the 100 year estimates is larger than that of the other case studies. The range of the *α*‐cut for *α* = 0 is 1.17, which is 32% of the midpoint of the cut. In contrast, the corresponding percentages of the Rhine and Werra case studies are 9% and 14%, respectively. Similar to the Rhine case study, the fuzziness increases with the return period as a result of the non‐fuzzy prior distribution and the inclusion of fuzzy historical information in the likelihood function.

Figure 11b shows that the imprecise credibility bounds are distinct for larger return periods, particular for the *α*‐cut for *α* = 1. Clearly, for this case study, the index estimates are both quite uncertain and imprecise due to a 300 year coverage of rather imprecise index values from the historical information.

## 5 Discussion

### 5.1 Value of Fuzzy Historical Information

The methodology proposed for transforming historical records into fuzzy numbers representing peak discharges of historical flood events, seems to be flexible in terms of being able to be adapted to diverse types of linguistic evidence and hydromorphological conditions. The evidence used ranges from descriptions of the water level to damage descriptions, and was drawn mainly from narratives and institutional archival records. This information was included in the likelihood function (Rhine and Tisza) or both the prior distribution and the likelihood function (Werra). The hydromorphological conditions range from an Alpine river with bedrock profile (Rhine) to a flat lowland river with extensive flood plains (Tisza) which, together with different linguistic evidence, resulted in different degrees of fuzziness.

In order to understand the stochastic uncertainty reduction due to historical information we defuzzify the fuzzy credibility bounds by taking the centroids of the membership functions. Table 6 evaluates the defuzzified 5% and 95% Bayesian credibility bounds of the cases shown in Figures 7b and 9b, and compares them with the credibility bounds that only use systematic discharge data. The range of the credibility bounds (shown in brackets) is a measure of the stochastic uncertainty associated with the estimates.

**Table 6:** Uncertainty of the Flood Quantiles for the Rhine and Werra Case Studies When Using Systematic Discharge Data Only and When Including Fuzzy Historical Information^a^

	Rhine at Basel			Werra at Meiningen		
	10 years	100 years	1000 years	10 years	100 years	1000 years
Systematic data only 5%–95% (range)	3355–3686 (330.7)	4223–5045 (821.7)	4817–6447 (1631)	160.8–189.2 (28.4)	210.2–288.6 (78.4)	237.9–359.3 (121.4)
Systematic and historical data (defuzzified) 5%–95% (range)	3370–3557 (187.2)	4481–4934 (453.1)	5347–6402 (1055)	165.4–183.2 (17.8)	220.7–251.5 (30.8)	248.2–322.4 (74.2)
Reduction in range	*−34.9%*	*−44.9%*	*−35.3%*	*−37.3%*	*−60.7%*	*−38.9%*

a The table shows the 5% and 95% Bayesian credibility bounds (m^3^/s) associated with the return periods of 10, 100 and 1000 years, their ranges as well as the percent reduction of the ranges when including historical information.

For the Rhine case study and a return period of 10 years, the range between the 5% and 95% credibility bounds decreases from 331 to 187 $m^{3}s^{-1}$ if fuzzy historical information is included. This conforms to a 35% reduction in the stochastic uncertainty. As the return period increases, the percent reduction slightly increases (to 45% for 100 years) and returns to 35% for 1000 years. The larger reduction for 100 years may be related to the typical magnitudes of large floods in the historical period. For the Werra case study the reduction in the stochastic uncertainty is slightly larger than for the Rhine (37%, 61% and 39% reductions for 10, 100 and 1000 years, respectively). The larger reduction is due to the shorter record length of the systematic sample (95 years for the Werra as compared to 142 years for the Rhine). Clearly, as the length of the systematic sample decreases, the historical information will become more important.

It is now of interest to compare the new fuzzy method with more traditional methods of including historical information that associate each historical sample with an uncertainty distribution (rather than with imprecision). Irrespective of the method used one would expect the uncertainty reduction to increase with the record length of the historical sample, decrease with the record length of the systematic sample, and decrease with the uncertainty of the historical data [*Francés et al*., 1994; *Hosking and Wallis*, 1986]. Using a similar Bayesian (but non‐fuzzy) framework, *Viglione et al*. [2013] found the range of the 5–95% credibility bounds of the 100 year flood to decrease by 60.9% when including historical data from a period of 350 years with a systematic record of 55 years in an Austrian catchment. For generated data, *Reis and Stedinger* [2005] found a reduction of 68% of the range of the 2.5–97.5% credibility bounds of the 100 year flood when including 100 years of historical data (without stochastic uncertainty) with a systematic record of 20 years. For a study set in southern France *Neppel et al*. [2010] found a 24% decrease in the stochastic uncertainty (150 years of historical data (without stochastic uncertainty) with a systematic record of 115 years). However, they also noted that there were cases when the uncertainty increased (between a 49% and a 69% increase of the range of the 5–95% credibility bounds of the 100 year flood) because of the high level of uncertainty affecting the highest historical floods and the model used for the systematic errors of the rating curve. While a formal comparison with alternative methods is beyond the scope of this study, the reductions we find here are consistent with the non‐fuzzy studies in the literature. In addition to the reduction in the credibility bounds, we account for the imprecision of the historical data and its propagation to the flood estimates. It has been checked that, in the limiting case where the fuzziness approaches zero (i.e., the membership functions collapse to a singe value and the fuzzy floods become crisp numbers), the quantiles obtained by the Fuzzy Bayesian Inference are identical to those of the Bayesian approach of *Reis and Stedinger* [2005] and *Viglione et al*. [2013]. In order to fully assess the accuracy of the presented method relative to other approaches, a cross‐validation including several combinations of historical and systematic periods in the sample, a comparison with other methods that use historical floods in the frequency estimation, and perhaps synthetic realizations where the flood samples are simulated with known properties, would be of interest.

### 5.2 Potential Biases

When estimating floods of a given return period one is equally interested in minimizing the biases as minimizing the uncertainty. Biases depend on the estimation method and the data used. The most critical issues when using historical, documentary evidence are probably observation biases, and in particular the difficulty of distinguishing whether, in a particular year, no flood has occurred or no flood has been reported even though a flood has occurred. The observation biases usually increase as one goes back in time because of the less systematic recording in earlier times and the increased likelihood that the information has been lost in the meantime. If information on the percentage of missed floods is available one can make adjustments by assuming that part of the years without observed floods have actually had a small flood. Information on missed floods can be obtained by comparative studies, e.g., by comparing archival information with measured discharges. However, this is only possible for the instrumental period while further back in history, when observational biases are likely larger, this is more difficult. Another possibility is to use the seasonal weather conditions from documentary evidence to assess the potential for observation biases, although this only gives a very approximate estimation. There are counterexamples where a drought was suddenly followed by a flood such as was the case in 1362 on the Hungarian Danube. More research is needed on this issue.

As noted by *Neppel et al*. [2010] and *Viglione et al*. [2013], the perception threshold is one of the most sensitive parameters in the estimation procedure. The perception threshold is usually set as the smallest flood discharge (or magnitude index) in the historical period, but this may imply missing floods above the threshold. Alternatively, as in the Rhine case study of this paper, a time varying threshold can be used. A time varying threshold can account for lower thresholds further back in history as is usually the case, but it does require more detailed information on the reliability of the historical sources over time and multiple subjective choices.

One of the motivations of using historical information in flood frequency estimation is the reduction in bias for the case the systematic period may happen to lie in a flood‐poor or in a flood‐rich period [*Hall et al*., 2014]. For example, for a study in the Ebro basin in Spain (300 years of historical data with a systematic record of 85 years), *Ruiz‐Bellet et al*. [2015] reported a 35–100% decrease in the best estimate of the 100 year flood when including historical data. However, this reasoning is based on the assumption that the historical period is more representative of the future than the systematic period. Flood‐poor and flood‐rich periods have indeed often been reported in the literature which are related to the low frequency variability of the hydrological cycle in the climate system [e.g., *Koutsoyiannis and Montanari*, 2007; *Schmocker‐Fackel and Naef*, 2010; *Szolgayová et al*., 2014]. Nonstationarity may also occur in the catchment and the river system, such as land use change, river training and diversions [*Hall et al*., 2014]. If known, such changes can easily be accounted for in the historical data as in the Rhine case study of this paper. If they are not known, they add to the uncertainty of the flood estimates.

### 5.3 Practical Applicability of the Method and Outlook

The main motivation of the proposed fuzzy Bayesian inference framework has been the perfect match to the nonprecise nature of linguistic information on historical floods as available in archives and other historical sources. However, this comes at the cost of the flood estimates being fuzzy‐valued, i.e., one obtains a range of best estimates, including a range for each credibility bound. This information may not be easy to use in practice, as practitioners tend to find it difficult to deal with stochastic uncertainty, let alone with both stochastic uncertainty and imprecision [e.g., *Blöschl*, 2008]. For some applications, such as flood design, one would probably defuzzify the estimates. The imprecision could be useful as additional information on deciding on design characteristics such as free boards. For other applications, such as risk mapping, one could in fact use the imprecision to produce fuzzy risk maps similar to uncertain risk maps [e.g., *Di Baldassarre et al*., 2010]. More generally speaking, imprecision could contribute to more informed decision making in a similar way stochastic uncertainty estimates do, even though stochastic uncertainty and imprecision are completely different concepts.

The proposed framework competes with alternative, traditional approaches of including historical information that are based on assigning stochastic uncertainty (rather than imprecision) to historical floods. While, conceptually, the fuzzy approach is appealing as it represents imprecision, in practical terms there is an element of similarity in that both approaches provide ranges for the estimates. *Stedinger and Cohn* [1986], *Francés et al*. [1994], and *Reis and Stedinger* [2005], e.g., consider historical floods as either perfectly known or as being larger than the perception threshold. *Botero and Francés* [2010] present a classification of censored historical information according to the exceedance of a perception threshold, and the presence of upper and lower bounds. In contrast, *Neppel et al*. [2010] and *Viglione et al*. [2013] consider a single historical flood as uniformly distributed between a lower and an upper threshold. This probabilistic approach could be extended to fuzzy‐valued distributions representing single historical events.

For the Tisza case study, magnitude frequency curves were estimated because it was difficult to estimate discharges for the flood events. While magnitude frequency curves cannot be used directly for flood risk management, they could contribute to enhance public awareness, and to put risk estimates (from other sources) into a qualitative long‐term context. Alternatively, discharges could be introduced in the analysis, but more information on the changes that the city and the catchment have undergone would be required.

Some of the assumptions of the proposed framework could be relaxed and some of the methods could be refined. For example, the simple threshold method used for the Werra case study to transform indices into discharges could be replaced by kernel density estimation [*Rosenblatt*, 1956] although preliminary tests (not shown here) suggest that the estimates thresholds did not differ by more than by 5%. Trapezoidal membership functions were used here, but other expressions could be used (e.g., general fuzzy numbers [*Viertl*, 2011b]). Future lines of research could include more formal sets of rules or mappings between textual descriptions of historical flood events and the corresponding fuzzy membership functions. Finally, the framework proposed here could be expanded to include regional and causal information in the spirit of *Viglione et al*. [2013].

## 6 Conclusions

This paper proposes a novel fuzzy Bayesian inference framework for flood frequency estimation that combines nonprecise historical flood information and systematic discharge observations. The following conclusions can be drawn from the application of the framework to the three case studies:

1. The proposed method is flexible in that it is able to account for different types of historical flood information as illustrated by three diverse case studies.
2. Depending on the historical source material, descriptions provide information with different levels of vagueness. These can be encapsulated in the membership functions with different widths for each flood.
3. Historical flood time series are usually given as three‐scaled magnitude index series. These lend themselves conveniently to be transformed into three‐parameter distributions.
4. Hydraulic reconstructions of discharges may have the narrowest membership functions. Their width can be based on the spatial extent of the flood waters reflected in the linguistic descriptions these reconstructions are built from.
5. If the historical and systematic records overlap, information on the historical floods can be included as a fuzzy prior distribution.
6. Inclusion of fuzzy historical information reduces the estimation stochastic uncertainty over only using systematic data. For the Rhine and Werra case studies, the stochastic uncertainty of the estimated 100 year flood was reduced by 45 and 61%, respectively.
7. If the historical flood records are not complete, observational biases are introduced. Similarly, if the catchment or river conditions change, biases are introduced. The latter can be easily accounted for if known, the former are more difficult to address.
